# An overview of the developments in 3D cancer cell models, assay techniques, and imaging modalities

**DOI:** 10.1007/s13577-026-01375-3

**Published:** 2026-04-04

**Authors:** Nicola H. Wheeler, Kayode A. Dada, Carrie A. Minnaar

**Affiliations:** 1 Department of Radiation Sciences, Faculty of Health Sciences, University of Witwatersrand, Johannesburg, South Africa; 2 Department of Mechanical Engineering Science, Faculty of Engineering and the Built Environment, University of Johannesburg, Johannesburg, South Africa

**Keywords:** Spheroids, Microphysiological systems, Organ design, Engineered aggregation, Microarchitecture

## Abstract

Cancer biology is a constantly evolving field of study due to the dynamic and complex nature of biological systems. The unique role of in vitro assays in cell biology has led to numerous transformations in our understanding of the functional and structural details of the cells and tissues that comprise these systems. However, the traditional monolayer assays have been reported to fall short of the in vivo physiology of cells. This has led to the development of 3D cell models, such as spheroids and organoids, that aim to recapitulate the intricate structural and functional behaviour of in vivo tumours. This review describes passive methods of spheroid formation (scaffold-free and scaffold-based) and the limitations that have driven the development of engineered active design methods to increase the physiological relevance of the model. Traditional assays need to be modified to evaluate these models, accounting for their architecture, density, and microenvironment gradients. Current developments in performing 3D cell assays include increased reagent concentration and incubation periods; however, many protocols still require single-cell analysis. We present a review of assay developments that maintain the spatial and contextual information that makes the 3D models physiologically relevant. Additionally, we introduce the advances in microscopy techniques that provide deeper visualisation of these models.

## Introduction

The transformations in cancer biology and treatment efficacy have long relied on in vitro models that mimic the in vivo behaviour and therapeutic responses of cancer cells. Traditionally, monolayer cell culture has served as the cornerstone for preclinical cancer studies due to simplicity, scalability, and cost-effectiveness. However, the lack of spatial architecture, tissue-specific heterogeneity, and dynamic cell-cell and cell-extracellular matrix interactions limit its clinical relevance [1]. This gap in physiological relevance has driven a shift towards more realistic models that can be translated into clinical cancer care. Recent advances mark a conceptual shift from the passive self-assembly seen in conventional 3D models towards ‘organ design’, where engineered intervention allows for the active and directed design of the organoid architecture by manipulating conditions, such as viscosity[2–4], scaffold stiffness[4], and oxygen permeability[5]. The first-generation spheroid model, relying on passive aggregation, is labour-intensive and time-consuming. Although these models effectively simulate the avascular features of solid tumours, hypoxia-induced necrosis and limited differentiation capacity make them inadequate in simulating the vascular phase of well-advanced tumours. Thus, the field of organ design was developed. Here, physical parameters, including mechanical stiffness, are engineered to drive rapid aggregation [4, 6, 7]; microchannel networks are utilised to inhibit necrosis [8]; and microphysiological systems (MPS) are utilised to create a dynamic environment that sustains growth and proliferation [5, 9–11]. Advancements in organ design necessitate the modification and optimisation of conventional viability and cytotoxicity assays to account for the complex microarchitecture of 3D models. New approaches to functional profiling are being adopted to assess behaviour. This review discusses the limitations of conventional spheroids and how purposeful design has enabled the creation of 3D models with organ-level physiology. Also, this review introduces the advancement of microscopy techniques that address challenges related to the inherent thickness and opacity of 3D models, and light penetration and scattering [12].

## Advancements in 3D cell models

The landscape of biomedical research has been transformed by the many advancements in 3D cell models, to bridge the gap between in vitro modelling and in vivo organ behaviour. Efforts to increase the complexity of MPS reveal a drive towards higher biomimetic fidelity and enhanced clinical translation. The evolutionary progression of these models can be characterised by the modular addition of specific in vivo features that were absent or limiting in previous, simpler systems.

### Spheroids

**Fig. 1:**
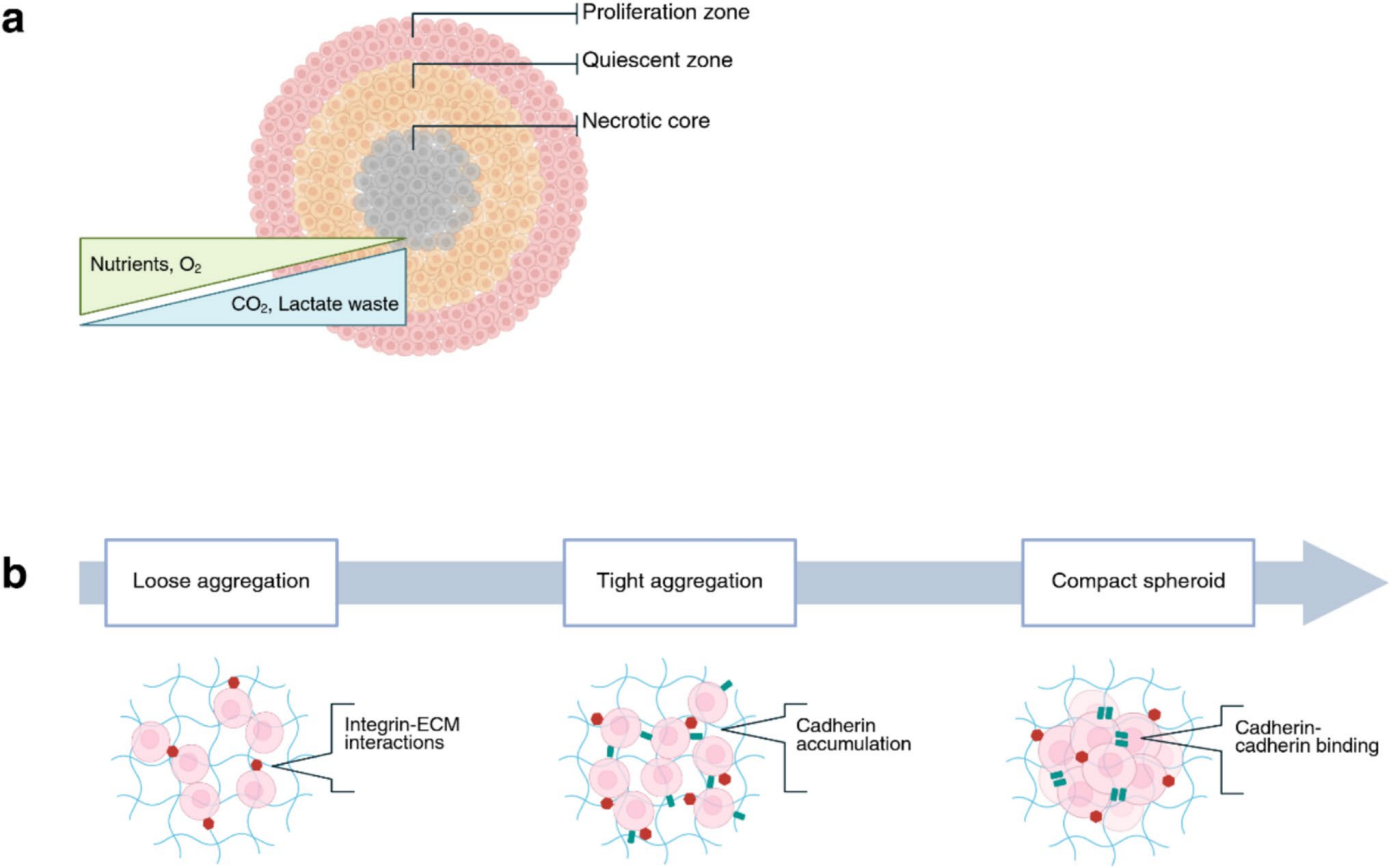
**Spheroid structure and growth.** **a** Three concentric layers of the spheroid. The outer rim is the proliferation zone, consisting of cells that have direct access to nutrients and oxygen and thus continue to actively divide. Moving towards the centre of the spheroid, oxygen and nutrient availability become limited, and in this quiescent zone, cells are viable but non-proliferative. In spheroids exceeding 200 μm in diameter, the necrotic core develops due to a severe lack of oxygen and nutrients, as well as the accumulation of metabolic waste. **b** Spheroid formation occurs in three phases. First, cells form loose aggregates through integrin-ECM interactions. Then, cadherin expression rises and accumulates, prolonging this phase of compaction. Finally, homophilic cadherin-cadherin interactions facilitate the formation of discrete and dense spheroids that will continue to grow. Created in BioRender. wheeler, N. (2026) https://BioRender.com/9nyjjxg

Spheroids represent the foundational step in the shift from 2 to 3D in vitro models, offering a simple yet effective method to restore the spatial context of native tissues through self-aggregation, a significant limitation of monolayer cultures. Spheroids are clusters of cells that self-assemble in physiological environments where intracellular adhesion, mediated by cadherins and integrins, is encouraged [13]. Formation occurs in three stages (Fig. 1B). As the spheroid grows, with a diameter exceeding 200 μm, steep oxygen and nutrient gradients develop, and a necrotic core can be observed in larger spheroids, 500 μm in diameter [14]. By mimicking critical features of poorly vascularised tumour regions, such as biochemical gradients [12, 15], intracellular communication [16], and zonation [13] (Fig. 1A), spheroids serve a variety of purposes in oncologic research; simulating drug penetration and studying chemoresistance [17].

Homotypic spheroids, usually formed from a single cancer cell line, provide high-throughput and consistency for early stage drug screening, but lack the cellular heterogeneity required to simulate the interactions within the tumour microenvironment (TME). By incorporating diverse cell types, such as cancer cells, fibroblasts, immune cells, and epithelial cells; the resulting heterotypic spheroid exhibits the stromal interactions, and immune components present in TME to better recapitulate the in vivo tumour and microenvironment. By incorporating cancer-associated fibroblasts (CAFs) into breast cancer heterospheroids, the mechanisms of CAF-driven cancer expansion and invasion could be elucidated [18, 19].

### Organoids

**Fig. 2:**
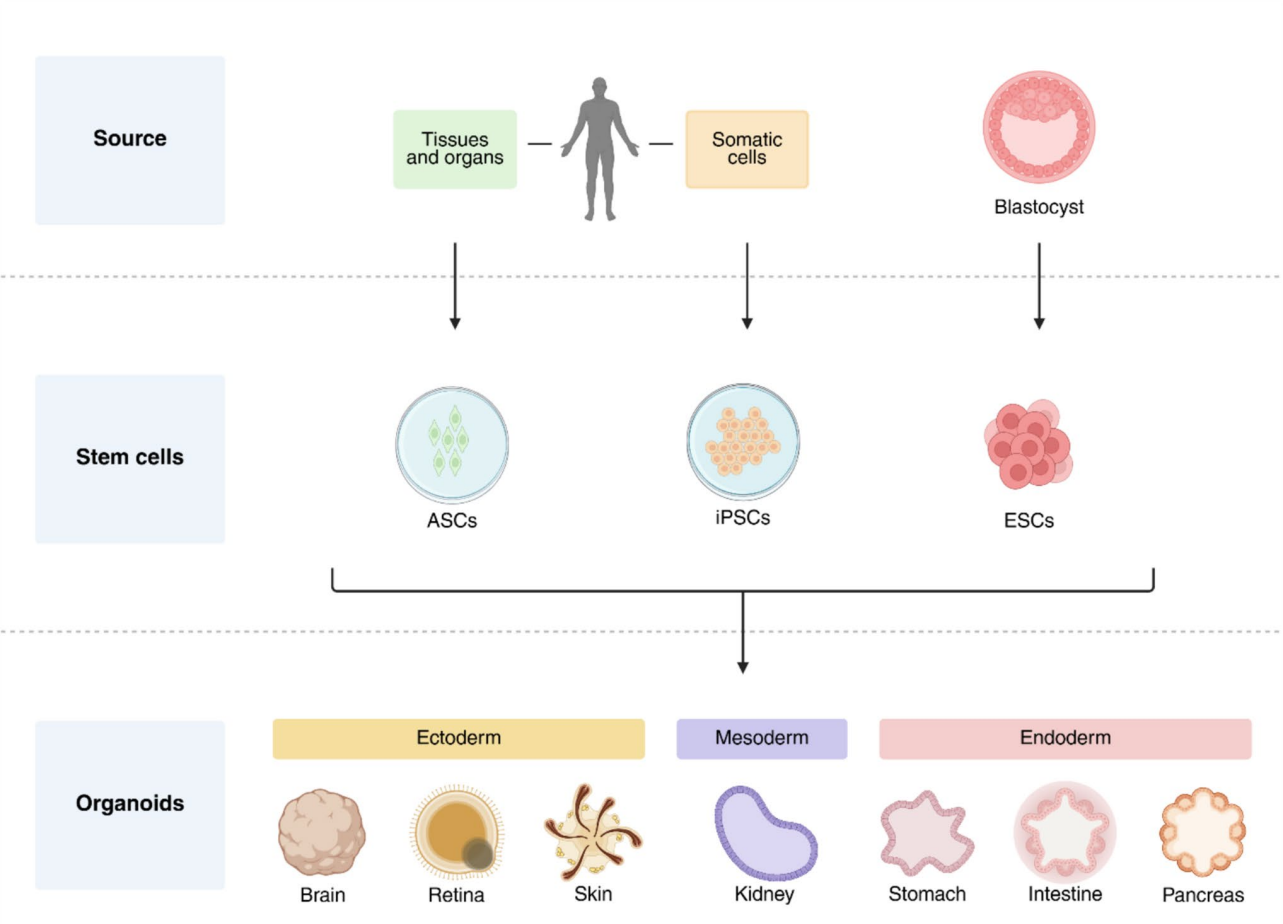
**Organoid development.** Unlike spheroids, organoids have self-organising and regenerative abilities, because they are derived from stem cells. Adult stem cells (ASCs), isolated from specific organs and tissues, produce mature organoids suitable for personalised disease modelling. Induced pluripotent stem cells (iPSCs) are isolated from somatic cells and reprogrammed with pluripotency-inducing transcription factors. This pluripotency enables the development of complex and multilineage organoids. Embryonic stem cells (ESCs) are selected for their ability to differentiate into various cell types and exhibit pluripotency. Created in BioRender. wheeler, N. (2026) https://BioRender.com/dp0hcnf

Organoids, having stem cell potential, recapitulate both the organisation and functionality of their tissue of origin [21] (Fig. 2). Whilst spheroids recapitulate tissue structure, organoids exhibit greater complexity and replicate the heterogeneity, structure, and native behaviour of the in vivo tumour due to their highly ordered and polarised nature. Successful generation and terminal-stage differentiation require spatiotemporal cues from growth factors, nutrients, and supportive matrices to stimulate the organ niche [22–24]. Although the complexity and physiological relevance have increased, organoids still relying on self-organisation; lack vascularisation, structural organisation, or environmental control. Sachs et al. established a breast cancer biobank of organoids to investigate subtype-specific resistance mechanisms, capture extensive inter-tumoral heterogeneity and mirror patient-specific response [25]. Extending into this workflow, Wang et al. demonstrated that ovarian PDOs are particularly useful in resolving discrepancies between static genomic biomarkers and actual therapeutic benefit, achieving 91.67% accuracy in predicting response to chemotherapy [26].

### Microphysiological systems

The use of microfluidic-based platforms produces the most advanced models which are referred to as microphysiological systems (MPS), or organ-on-a-chip (OOC). The tunability of OOC platforms allows for enhanced control of the fluid flow, pH, glucose, and oxygen levels to more accurately mimic the cellular microenvironment and maintain physiological functions [27]. Continuous fluid perfusion within the microchannels simulates key aspects of blood (and other physiological) flow. Shear stress and mechanical strain provide the necessary cues for functional maturation, and continuous perfusion ensures effective nutrient circulation and waste removal.

Recent breakthroughs in liver microphysiological systems highlight the necessity of integrating endothelial barriers and dynamic fluid flow to achieve the level of biomimicry required for accurate clinical predictions [28–30]. Microperfusion of mesoscale liver cultures established physiologically relevant oxygen gradients and metabolic zonation required for disease studies [29]. These advances can serve as the foundational blocks for building “liquid liver” models that will focus on reversing dysfunction-associated steatotic liver disease.

## Methods of forming spheroids

Currently, there is no singular standardised method for generating 3D spheroids, as various methods have been established to produce these models. Traditionally, spheroid generation relies on spontaneous self-assembly of aggregates, including scaffold-free approaches such as hanging drop [6, 13] and low-attachment plates [14], and scaffold-based techniques that use hydrogels to provide structural support and mimic the extracellular matrix [15].

### Scaffold-free methods

Widely recognised for its simplistic approach and cost-effectiveness, the hanging-drop method relies on gravity to encourage cells, suspended in an inverted drop of medium, to self-aggregate into spheroids [15, 31]. This platform is limited by spherical geometry, risk of evaporation, and low throughput [32]. Tung et al. developed a versatile 384 hanging-drop array plate that incorporated access holes on the top of the surface of the plate, enabling more stable culturing conditions and simplified media changing procedures [15].

The liquid overlay technique is favoured for its reproducibility and accessibility [14]. Whilst this technique has successfully established gastric cancer spheroids, with 12 of 17 cell lines recapitulating the differentiation phenotype of parental carcinomas [17], it is labour-intensive. To address the limitations of size and cell composition heterogeneity, the addition of centrifugation has improved uniformity [33]. However, it remains time-consuming and labour-intensive. Sun et al. developed a four-inlet flow-focussing microfluidic device [11] that enables the fabrication of approximately 200 highly structured spheroids per minute. The fabrication of size-controlled spheroids with high sphericity is a challenge of heterotypic spheroid fabrication using the conventional liquid overlay technique; however, the flow rate of the continuous oil phase of the microfluid system can be manipulated to control size [11].

Rotating bioreactors provide an enhanced, stimulating growth environment suitable for coculture to generate heterotypic spheroids. As cells in suspension are continuously moved, the spinning speed needs to be monitored to minimise shear stress exerted on the cells and prevent cell damage [34]. The high surface-to-volume ratio makes this technique favourable for culturing larger tissue architecture, as continuous medium mixing provides effective nutrient and waste transport, and uniform distribution of oxygen [34].

Regardless of the method chosen, control of spheroid size and uniformity is a continued challenge in the conventional spheroid culture. The methylcellulose (MC) medium uses phase separation and dehydration forces to yield cell aggregates within minutes, addressing the long culturing process that limits hanging drops’ utility. This high-molecular-weight cellulose derivative has been used as an ECM-mimicking scaffold for the rapid aggregation of spheroids [4, 6], to support the consistent formation of size-controlled and uniform spheroids [18], and by suppressing diffusion, endogenous ECM proteins are retained [35]. When dissolved in cold water, polymer chains within the MC structure are hydrated, forming a highly viscous solution. When cells are injected into MC-supplemented medium, MC’s swelling property allows it to absorb water and the normal medium, leaving the suspended cells trapped at the interfaces. Cell movement is restricted, cell-cell interactions are increased, and rapid aggregation is forced. The degree of aggregation is determined by the MC concentration [6]. The MC medium provides a semi-solid matrix for cells, mimicking in vivo extracellular geometry whilst promoting tight connection between collagen and integrins to support 3D growth [36]. Ware et al. found that the addition of MC to the hanging drop method produced pancreatic spheroids that expressed Ki-67 and HIF-α levels comparable to those expressed in xenograft models [37].

### Scaffold-based methods

For scaffold-based methods, natural (collagen, elastin, and fibrin) and synthetic polymers [polyethene glycol (PEG) and polyvinyl alcohol (PVA)] can be used to fabricate ECM-like structures that replicate the structural and biochemical properties of the in vivo TME. To encapsulate the glioma microenvironment, a 3D scaffold can be fabricated by cross-linking chitosan with gelatine [38]. MCF-7 cells cultured in this matrix demonstrated increased cancer stem cell expression and tumorigenesis due to increased pro-angiogenic growth factor and matrix metalloproteinases (MMPs) expression [38, 39]. The proliferation patterns of these spheroids resemble those of in vivo tumours [39]. The incorporation of natural compounds in scaffolds presents considerable challenges for consistency and reproducibility in spheroid generation due to the inherent variability of their biochemical properties [40]. Whilst synthetic polymers allow control and customisation of their biochemical properties, surface modifications and coatings are required to impart bioactivity. Thus, consideration of polymer concentration is necessary, as this parameter significantly influences the architecture of the resulting scaffold [41, 42].

Matrigel, a solubilised basement membrane preparation, effectively mimics the real, native ECM and is widely used as the foundational material for 3D culture. Matrigel is derived from the extract of basal membrane proteins present in the Engelbreth-Holm-Swarm (EHS) mouse sarcoma line [43]. It contains a mixture of structural proteins (laminin, collagen IV, and entactin) and growth factors (TGF and EGF), allowing for the development of spheroids and organoids characterised by complex architecture and dimension [1]. At physiological temperatures, the protein content in Matrigel polymerises to form a stable hydrogel that provides a scaffold for structural support and biochemical signals necessary for cellular proliferation and differentiation. The laminin and collagen in the ECM promote cellular attachment. Whilst considered the ‘gold standard’ of reconstituted ECM, the steep price tag and ill-defined, animal-derived composition, causing variability, have encouraged the development of collagen biomaterials [44].

In a three-step process, Takezawa et al. [2] developed an ECM-based matrix with high mechanical strength [2] and stability, called collagen vitrigel (CV). Formed from purified type 1 collagen, a well-defined material that is the primary component of desmoplastic stroma in solid tumours [18], CV eliminates the uncertainty of non-biological variance associated with Matrigel. The vitrification process ensures that every membrane produced has uniform thickness, fibrillar density, and transparency, providing a scaffold for regenerative medicine applications, such as corneal regeneration [3]. Maintaining spheroid integrity and preventing disintegration during media replenishment and handling are concerns for many passive techniques; however, the vitrified collagen membrane can be easily manipulated whilst keeping spheroids intact. The addition of CV to hepatic spheroid cultures maintained enhanced albumin secretion, urea synthesis, and structural stability for at least 3 weeks [9, 31]. Maintaining metabolic functions for prolonged periods is vital for anti-cancer drug toxicity testing. Additionally, CV has proved to be an ideal matrix for reconstructing organoids [2] by promoting proliferation, by 68% in liver organoids compared to those cultivated in traditional collagen scaffolds, and supporting the formation of polarity in epithelial cells.

## Active design: engineered control of spheroid architecture

### Engineering architectural complexity and morphogenetic control

Biomechanical properties, such as stiffness and viscoelasticity, and biochemical cues can be engineered into hydrogel scaffolds and microparticles to guide cell behaviour and tissue fusion. The ECM continually undergoes remodelling through synthesis and degradation mechanisms [7, 18, 20, 45]. The surrounding collagen matrix creates a physical barrier, thus limiting drug penetration and accumulation within the spheroid core [45]. Lo Cicero et al. demonstrated that incorporating an endogenous ECM into 3D models is vital for the development of chemotherapeutics, as the failure of anti-cancer drugs may not be solely attributed to cellular resistance but also to physical exclusion from the remodelled TME.

The structural density and rigidity of a collagen matrix directly influence the assembly of the actin cytoskeleton, which in turn regulates cell morphology and function. In high-density matrices, such as the CV membrane, a stiff in vitro tumour environment is created, leading to CAF activation [18]. The spatial arrangement of tumour cell clusters within the TME, or inter-spheroid proximity, is a critical determinant of invasion kinetics [18]. In engineered 3D environments, the distance between spheroids influences the intensity of mechanical crosstalk. Through this mechanism, CAFs communicate across the fibrous matrix by sensing and responding to the traction forces exerted by neighbouring cells [18, 45].

Kojima et al. established a method for incorporating microchannel networks in spheroids, enhancing nutrient exchange [8]. Heterospheroids containing HepG2 cells and alginate hydrogel beads in an irregular arrangement were rapidly formed using the MC medium. The presence and connectivity of the microchannel network were confirmed by fluorescence staining in almost all areas of the hetero-spheroid, compared to the minimal staining seen in the conventional spheroids, indicating that the engineered microchannels allowed complete access from the surface to the core of the hetero-spheroid [8].

### Conquering the diffusion limit: mass transport and metabolic homeostasis

Although the necrotic core is useful for modelling metastasis and immune cell studies, it can interfere with the accuracy of drug toxicity screening and drug penetration studies. In passive methods, the number of cells seeded is controlled to prevent spheroids’ exceeding 300 μm and developing necrosis [46]. However, leaving the development of the core to spheroid size is not ideal, and so many groups have investigated the oxygenation of MPS to inhibit hypoxia-induced necrosis.

Applying a gas-permeable material, polydimethylsiloxane (PDMS), to culture vessels enables direct, continuous oxygen supply to growing spheroids, but controlling spheroid size is challenging [5]. Size is a factor in oxygen mass transfer, and so, Anada et al. fabricated an oxygen-permeable chip (oxy chip) using PDMS with multiple cavities enabling controlled spheroid growth [5]. Interestingly, the oxy chip dramatically inhibited hypoxia in the core of spheroids despite their diameter exceeding 200 μm, the diameter limit for preventing necrosis in conventionally formed spheroids. Using the oxy chip, the hypoxic and survival threshold diameters can be increased to 400 μm and 600 μm, respectively [5]. Whilst systems designed to enhance spheroid oxygenation aim to inhibit hypoxia-induced cell death [5, 8], the impact these methods have on metabolic performance must be discussed. The oxy chip cultivated spheroids that produced significantly less lactate and consumed significantly less glucose than conventional spheroid and monolayer cultures indicating that oxygenation improves cell function by altering glucose metabolism.

### Orchestrating biological maturation and phenotypic fidelity

The development of phenotypic function is heavily dependent on culture duration, as time is needed to establish cell-cell contacts, deposit ECM and maturate into functional in vivo-like tissues [47]. Spheroid formation occurs within 1-3 days, maximum phenotypic expression and metabolic activity develop over a longer period, 7-21 days. In liver spheroids (HepG2), achieving time-dependent metabolic maturation to functional levels comparable to those of mature liver tissue presents unique challenges. The design of a collagen vitrigel membrane chamber culture system enabled Watari et al. to maintain high levels of CYP activities in HepG2 spheroids for 21 days [9]. The addition of CV to the culture system established a platform for high prediction accuracy during toxicological assessment of test compounds.

Bioprinting technology is utilised in tissue engineering processes to fabricate functional tissue constructs [19]. Cells, spheroids, and biomaterials are precisely deposited into pre-defined 3D structures that mature into an in vitro model that accurately mimics the in vivo target tissue. This technology was employed to fabricate tumour-tissue constructs with differing tumour cell and fibroblast configurations [19] and the study found that cell configuration and design of model significantly impact tissue formation and maturation.

### Inducing barrier function and polarity

Advances in OOC platforms, are increasingly focussed on enhancing complexity and establishing organ-specific barriers, such as vascular [10] and intestinal [48–50] and blood–brain [51] barriers, to enable a deeper understanding of the protective and regulatory mechanisms present within the native organ niche. The establishment of functional barriers in organ models creates apical and basolateral sides, crucial for studying anti-cancer drug absorption and tolerance enabling more predictive preclinical [10, 49, 50].

Wang et al. developed a tumour-vascular barrier model by fabricating coculture spheroids in the lower chamber and culturing HUVECs in the upper chamber of the Transwell system [10]. Whilst the presence of the endothelial barrier in co-cultured, spheroid led to an increase in drug tolerance and cell viability, this system relies on diffusion between compartments for nutrient and drug exchange. The integration of perfusion in organ models to reproduce flow-induced shear stress, not only promotes cell morphology and polarisation [48, 49], barrier maturation, and functionality is enhanced [49]. By incorporating a perfusable hydrogel channel into a gut-on-chip microfluidic device, Vera et al. were able to reproduce the spatial organisation of the intestinal mucosa, by supporting the encapsulation of both epithelial and stromal compartments [49].

## Advancements in methods to investigate spheroid function and behaviour

As the cell models used in biomedical research and drug-screening applications become more complex, the traditional assays used to probe them need to be modified to account for increased size and density, spheroid compactness, and physiological gradients.

Many studies investigating cell viability and metabolic activity have successfully modified assay protocols to address dye diffusion and retention challenges [52–55]. Resazurin-based assays (AlamarBlue and PrestoBlue) simply require increased reagent concentration and incubation periods for sufficient spheroid staining, and 10% reagent concentration incubated for 2 h achieved reduced background noise during imaging [52, 53]. The CellTiter-Glo™ 3D reagent stands out for its superior accuracy, consistency, and efficiency. Its robust formulation ensures thorough spheroid lysis and ATP recovery regardless of cell density and hydrogel composition [56]. Notably, this assay is performed with the addition of a single reagent, eliminating the need for washing and medium removal [57]; maintaining spheroid structure and integrity.

Whilst these modifications and formulations allow for effective dye penetration and retention, these assays provide a binary assessment of cellular health, failing to reflect the functional status of the 3D model and hence fail to bridge the gap between in vitro findings and clinical outcomes.

The characterisation of the cell barriers established in the microfluidic devices used to rely on permeability and immunostaining assays [49]; however, these methods do not enable fast readouts. Additionally, interference of the fluorescent probes with the transport and diffusion of molecules in the MPS is a concern [50]. The transepithelial/endothelial electrical resistance (TEER) measurement has emerged as a highly sensitive, non-invasive technique for monitoring barrier integrity in real time [10, 27, 48–51, 58, 59]. The strength of the intercellular tight junctions and permeability can be measured without disrupting spheroid integrity. Electrodes to measure TEER can easily be integrated with the with various microfluidic systems [10, 27, 48–51]. The TEER measurement is used to quantitatively confirm the development of the barrier and is functionality [27, 49, 50]. Additional immunostaining (VE-cadherin) can be done to achieve visual confirmation of the structural integrity of the tight junctions [10, 51]. Furthermore, this measurement offers distinct benefits over traditional fluorescent probes. First, this measurement demonstrates the ionic conductance of both the paracellular and transcellular route of the epithelium, and second, it is an electrical resistance measurement, so diffusion interference concerns are eliminated [50].

## Visualising the 3D cell model

A fundamental concept in 3D geometry is the *z*-plane, which represents depth and allows for cellular structures to be visualised with spatial relationships and volume. The 3D structure is reconstructed with a series of ‘*z*-stacks’, which are a series of 2D microscope images captured at different focal planes along the vertical axis of a sample [60, 61]. There have been remarkable advancements in 3D imaging, summarised in Table 1, including the development of several microscope techniques to improve image resolution and penetration depth, and prevent cell damage.

**Table 1:** Key microscopy techniques for imaging 3D cellular structures

Technique	Principle	Imaging/penetration depth	Advantages	Limitations	References
Wide-field microscopy	A light source uniformly illuminates the entire field of view. 3D imaging is achieved by acquiring a series of 2D images along the z-stack	~ 200 nm	Low cost and accessible Large field of view Low phototoxicity	Poor axial resolution for larger models Z-stack acquisition is time-consuming Lack of optical sectioning Limited sample thickness	[60, 70]
Confocal microscopy	A single beam is used to illuminate a small volume of the sample, and the complete image is reconstructed one point at a time, whilst the out-of-focus signal is eliminated by a pinhole	60–100 μm	High-resolution. Optical sectioning improves z-resolution Out-of-focus light rejection Non-invasive	Slow imaging rates Expensive equipment Photobleaching and phototoxicity	[12, 62, 63, 68]
Light-sheet microscopy	A thin sheet of laser light illuminates a specific plane within the cell model. Optical sectioning is inherent, as only the fluorophores inside the light sheet are excited, and thus, no out-of-focus light is created	200–400 μm	Good depth penetration High speed High resolution Low phototoxicity and photobleaching	High cost Scattered background light	[67, 68]
Multiphoton microscopy	Longer-wavelength near-infrared pulsed lasers excite fluorophores that simultaneously need to absorb two or more lower-energy photons and emit a single, higher-energy visible photon. This nonlinear excitation is confined to the focal point	250–500 μm	Good depth penetration Reduced scattering Deeper imaging High resolution	Specialised equipment is needed Long imaging time Phototoxicity with high peak excitation intensity	[65, 66]
Optical coherence tomography	Uses low-coherence light to create cross-sectional images based on reflected light	~ 500 μm	High structural resolution High acquisition speed Non-invasive Minimal phototoxicity Label-free	Limited penetration depth The opacity of the interfering medium affects optimal imaging	[69, 71–73]

Confocal laser scanning microscopy (CLSM) is the most common confocal microscopy technique, providing better background rejection and sharper images [12]. However, the point-scanning nature of this method leads to slow image acquisition and increased phototoxicity and photobleaching [62, 63]. The combination of scanning electron and confocal microscopy provides detailed illustration of morphological changes of spheroids treated with cytoskeleton-disorganising agents [64]. Phillips et al. established a method of embedding spheroids within collagen hydrogels, preventing inconsistent placement in the *z*-axis and enabling consistent high-resolution imaging in confocal, multiphoton, and super-resolution spinning disc microscopy [62] Utilising CV in 3D cultures improves visualisation of the cells within the 3D matrix due to the transparency and clarity of the membrane.

Like confocal microscopy, Multiphoton Microscopy (MPM) eliminates out-of-focus light to allow for optical sectioning; however, it achieves twice the penetration depth of confocal [65]. Additionally, MPM captures multiple intrinsic contrasts from cellular biomolecules as well as second-harmonic generation contrast from non-labelled structures such as collagen, thereby enabling the visualisation of the ECM surrounding the cells [62, 66].

A specific type of LSFM, selective plane illumination microscopy (SPIM), is a powerful method for volumetric imaging of 3D spheroid cultures at single-cell resolution. SPIM has been successfully implemented to record all phases of spheroid formation in HepaRG and H2B-GFP hepatic models [67] and monitor the fluorescence signal of the whole spheroid, without loss of resolution [68].

Huang et al. demonstrated the reliability of optical coherence tomography to identify necrotic regions in multicellular tumour spheroids [69], whilst the limited depth penetration of traditional techniques limited the resolution of the entire structure.

## Conclusion

The foundation of cancer research has long been built upon in vitro 2D cell culture. However, these models, failing to reproduce the complexity of in vivo tissues, are a primary source of failure in clinical translation. The evolution from flat monolayers to complex 3D architectures represents a critical, necessary paradigm shift to bridge the gap between laboratory findings and clinical efficacy. The increasing complexity of 3D models necessitates a parallel evolution in the assays used to probe them. Additionally, there is a need to develop assays that study 3D cells in their native state, without disaggregating them into single cells for in vitro analyses. A dense, multi-layered spheroid or organoid is an optically opaque and light-scattering environment, rendering conventional microscopy redundant. Imaging these models requires advanced techniques to see deeper, faster, and more gently through the layers. The trajectory of preclinical cancer research is one of escalating complexity, driven by the fundamental need to create in vitro models that more accurately predict clinical outcomes. The journey from 2D monolayers to simple spheroids, and now to patient-derived organoids, marks a significant advance in preclinical studies.
